# Dermoscopy Differentiates Guttate Psoriasis from a Mimicker—Pityriasis Rosea

**DOI:** 10.5826/dpc.1101a138

**Published:** 2021-01-29

**Authors:** Meena Makhecha, Tishya Singh, Yasmeen Khatib

**Affiliations:** 1 Department of Dermatology, HBT Medical College & Dr. R.N. Cooper Hospital, Mumbai, India; 2 Department of Pathology, HBT Medical College & Dr. R.N. Cooper Hospital, Mumbai, India

**Keywords:** guttate psoriasis, pityriasis rosea, dermoscopy, mimicker

## Abstract

**Background:**

Guttate psoriasis (GP) and pityriasis rosea (PR) are a part of papulosquamous disorders that have very similar clinical features and often require histopathology to confirm the diagnosis. Dermoscopy has emerged as a noninvasive, cost-effective technique that can aid in the diagnosis of inflammatory skin diseases like GP and PR.

**Objective:**

To study and compare the dermoscopic features of GP and PR.

**Methods:**

Twenty consecutive patients each with GP or PR were enrolled in the study. The diagnosis of GP and PR were made clinically and on histopathology. Dermoscopic images were taken of the representative lesions from each patient using a manual dermoscope attached to a digital camera after applying ultrasound gel. Vascular morphology, vascular arrangement, background color, along with color and distribution of scales were noted in each case. Statistical analysis was done using chi-square test to determine the significance of findings in both groups.

**Results:**

The combination of a bright red background with dotted vessels in uniform diffuse distribution with diffuse white scales was highly specific for the diagnosis of GP. Lesions of PR showed a red background with dotted blood vessels in nonspecific distribution. Scales were either white in color or brown pigmented with patchy distribution. Brown pigmentation and brown dots were additional findings in cases of PR.

**Conclusions:**

Combinations of dermoscopic patterns can aid in the diagnosis of GP and PR in the majority of the cases.

## Introduction

Guttate psoriasis (GP) and pityriasis rosea (PR) have overlapping clinical features and pose a diagnostic challenge to dermatologists. It is important to differentiate these conditions, as they differ in clinical course, treatment, and prognosis. While PR is a self-limiting condition, psoriasis is associated with a myriad of complications and the course of disease is longer [1]. Histopathology has always been considered the gold standard to confirm the diagnosis but has the disadvantage of being invasive. Dermoscopy offers an alternative, quick, and noninvasive technique which can aid in diagnosis [2,3]. Studies have been done to illustrate the dermoscopic features of various papulosquamous disorders. However, studies comparing the dermoscopic features of PR and GP are lacking. Hence, this study was undertaken to analyze and compare the various dermoscopic features of both these conditions that in turn can help differentiate them from one another.

## Methods

The study was conducted in the Department of Dermatology at a tertiary care hospital in Mumbai. It was a cross-sectional, single-center descriptive study. Institutional ethics committee approval was sought before commencing the study. Twenty consecutive patients each of either GP or PR who attended the Department of Dermatology were enrolled in the study on the basis of inclusion and exclusion criteria after taking informed consent. Inclusion criteria consisted of all male and female patients of all age groups with GP and PR attending the Department of Dermatology of our institute. Patients on topical or systemic treatment within the previous month of recruitment were excluded from the study. The duration of the study was 3 months, from May to July 2019. A complete history and demographic profile of each patient was recorded followed by a clinical examination. The diagnosis of GP and PR was made clinically and confirmed with histopathology. Dermoscopic images were taken of the representative lesions from each patient using a manual dermoscope (Heine Dermatoscope; ×10) attached to a digital camera (Canon 1200 D DSLR) after applying ultrasound gel with minimal pressure over the lesion. Each dermoscopic image was then reviewed by 2 dermatologists. Concordance between dermatologists was considered when both the dermatologists agreed on the dermoscopic structure.

Variables used in the dermoscopic evaluation were (1) vessel morphology (dotted, linear); (2) vessel distribution (uniform, unspecific); (3) background color (bright red, dull red, skin-colored, hyperpigmented); (4) scale distribution (patchy, diffuse, central, peripheral); (5) scale color (white, brown pigmented); and (6) pigmentation (brown dots, diffuse structureless brown pigmentation) [4]. For each lesion a dermoscopic–histopathological correlation was performed.

## Statistical Method

After the data collection, data was analyzed using Microsoft Office Excel 2007 and SPSS Version 16.0. The data was presented as frequency and proportions. Chi-square test was applied and P value was calculated. A P-value of less than .005 was considered significant.

## Results

This study included 20 patients each with GP and PR. The maximum number of patients with GP and PR were in the age group of 20–30 years of age, 9 (45%) and 10 (50%) respectively. Twelve (60%) patients with GP and 11 (5%) patients with PR were males. Twelve (60%) patients with GP had trunk involvement; 5 (25%) had upper limb involvement, and 3 (15%) had involvement of the lower limbs. The figures for patients with PR were 14 (70%), 5 (25%), 1 (5%) respectively. The mean duration of GP was 4.2 +/− 1 week, and the mean duration of PR was 2.3 +/− 1 week.

The specific dermoscopic findings for GP and PR are summarized in Table 1. Red dotted vessels were seen in all 20 (100%) cases of GP. There was a uniform distribution of vessels in 17 (85%) cases. Thirteen (65%) patients with PR had dotted blood vessels. No other morphology of blood vessels was seen. There was an unspecific distribution of vessels in 12 (60%) patients (Table 1). Statistical analysis showed that the difference in the vessel distribution between the 2 groups and had a P value < 0.05, which was significant.

The bright red background was present in 11 (55%) of GP lesions and dull red background was seen in 9 (45%). In PR, bright red and dull red background was found in 7(35%) patients each (Table 1). The P value for background color between the 2 groups was 0.068, which was not significant. In 18 (90%) cases of GP, scales were present in a diffuse pattern and in 7 (35%) cases of PR either peripherally or centrally.

All the scales in GP were white in color; 11 (55%) cases of PR presented with white scales, and the remaining 9 (45%) cases of PR had brown pigmented scales (Table 1). P value was < 0.05 for the difference in scale distribution and scale color, which was significant.

Pigment pattern was present in the form of brown dots in 11(55%) PR patients while 5(25%) of the patients had diffuse brown pigmentation. In patients of GP, no pigmentation was seen (Table 1). Hence, the pigment pattern was significantly different between the 2 groups with the P value of < 0.05, which was significant.

## Discussion

**Figure 1:**
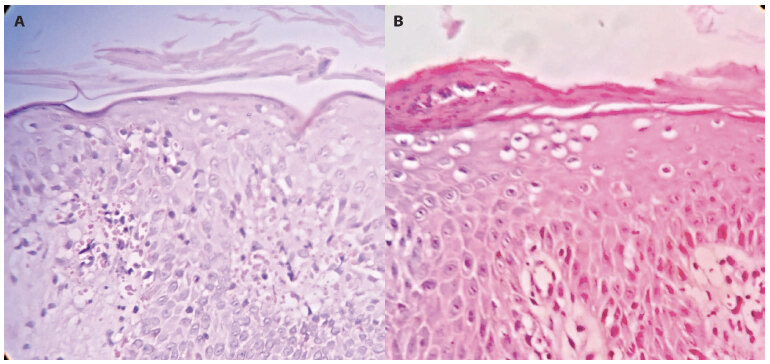
(A) Histopathology of guttate psoriasis shows dilated tortuous capillaries in the papillary dermis along with mononuclear and neutrophilic infiltrate. There is parakeratosis, with epidermal hyperplasia and rete ridges of even length (H&E, ×10). (B) Histopathology of pityriasis rosea shows focal parakeratosis, irregular acanthosis, spongiosis and exocytosis. A few necrotic keratinocytes along with extravasated red blood cells can be seen in the epidermis. The dermis shows superficial perivascular chronic inflammatory infiltrate with extravasated red blood cells in the papillary dermis (H&E, ×10).

Guttate psoriasis clinically presents with multiple erythematous papules to plaques with a fine scaling on its surface predominantly on the trunk and lower extremities. On histopathology, the lesions show dilated tortuous capillaries in a regular fashion in the papillary dermis along with mononuclear and neutrophilic infiltrate. There is parakeratosis, with epidermal hyperplasia and rete ridges of even length (Figure 1A). The correlation of these histopathological features with dermoscopic findings is presented in Table 2.

**Figure 2:**
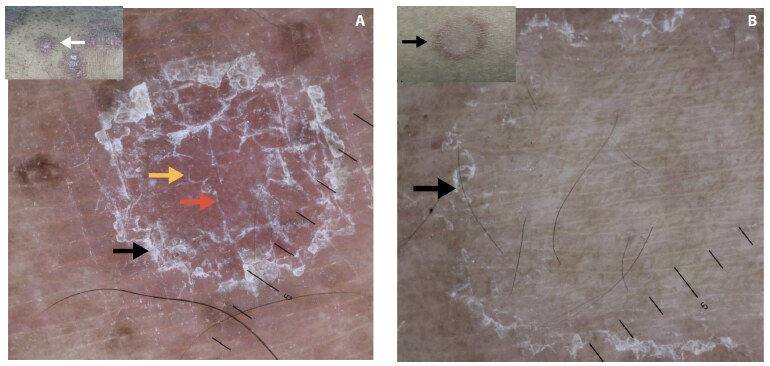
(A) Guttate psoriasis; clinical and dermoscopic pictures in the same patient. (A, inset) The white arrow indicates the lesion selected for dermoscopy. Dermoscopic image of the lesion reveals bright red background (yellow arrow) with diffuse scaling (black arrow) with a uniform distribution of blood vessels (red arrow). (B) Pityriasis rosea; clinical and dermoscopic pictures in the same patient. (B, inset) The black arrow indicates the lesion selected for dermoscopy. The dermoscopic picture reveals peripheral scaling (black arrow), as seen in pityriasis rosea, and a skin-colored background.

In the GP patients, dotted blood vessels are seen in 100% with 85% of patients having vessels in a uniform pattern. Dilated tortuous capillaries in the papillary dermis on histopathology show a very specific pattern on dermoscopy as regular, diffuse distribution of red dots, also seen by Errichetti et al [5]. A bright red background was present in 55%, whereas dull red background was present in 45% of patients (Figure 2A). The red background can be attributed to increased vascularity in the papillary dermis.

It was observed that, as the lesion starts becoming chronic, the erythema becomes dull and the distance between the red dots increases. This can be attributed to the increased epidermal proliferation present in the form of increased acanthosis as the disease progresses. Scales were present in a diffuse distribution in 90% and all (100%) were white-colored (Figure 2A). This diffuse distribution of scales on dermoscopy is attributed to the confluent parakeratosis on histopathology.

Clinically PR presents as erythematous brownish colored papules to plaques with an overlying collarette of scale and is commonly found on the trunk. The histopathology is nonspecific and shows focal parakeratosis, irregular acanthosis, spongiosis, and exocytosis [6–8]. A few necrotic keratinocytes along with extravasated red blood cells can be seen in the epidermis [9]. The dermis shows a superficial perivascular chronic inflammatory infiltrate with extravasated red blood cells in the papillary dermis (Figure 1B). The histopathological features with dermoscopic findings could be correlated (Table 3).

**Figure 3:**
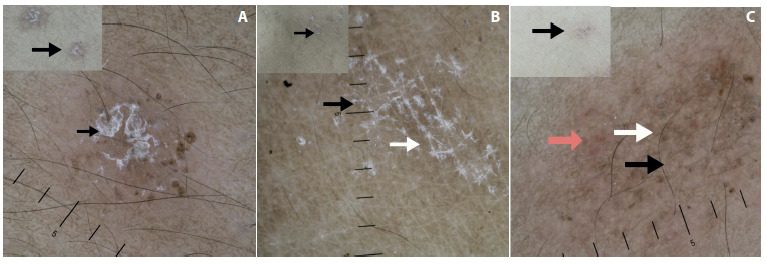
Pityriasis rosea. (A, B, and C insets) The black arrows indicate the clinical lesions. The dermoscopic image in: (A) reveals a central brown pigmented scale (black arrow); (B) a hyperpigmented background (white arrow) with diffuse white scaling (black arrow); and (C) diffuse brown dots (white arrow) with diffuse brown pigmentation (black arrow) and unspecific red dots (red arrow).

In patients with PR, blood vessels were observed in 65% patients, all of which were dotted and its distribution unspecific in 60% (Figure 3C). This is in accordance with previous studies stating that dotted vessels in PR lack the regular distribution of psoriasis [10,11]. Bright red and dull red backgrounds were both seen in 35% cases. Similar results were seen in another study: 25% of the lesions of PR had a dull red background, 10% of the lesions had a light red background, and 65% of the lesions had a yellow background [10]. The yellow background seen was not observed in the present study, although a skin-colored background in 20% of the lesions (Figure 2B) and a hyperpigmented background in 10% of the lesions could be appreciated (Figure 3B) that might be because of the difference in the Fitzpatrick color types among the subjects of both studies.

Another observation of this study was that, in early lesions of PR the scales were present more centrally; in old lesions, and in the herald patch the scale progressed towards the periphery. In 35% of patients with PR, a collarette of scale was present in the central region (Figure 3A) and an equal number of cases had a peripheral distribution (Figure 2B). One particular study states that central scaling was present in 5%, diffuse scaling was present in 15%, and peripheral scaling was present in 70% of lesions [10]. The difference in the findings of both these studies might be due to the selection of lesions [11], which may have been in the later stages of evolution. The present study had an equal number of patients in early and late stages of evolution.

Brown pigmented scales (Figure 3A) were present in 45% of patients, and white scales were present in 55% of patients (Figure 2B). Regarding scale color, white scales were evident in 85% and yellow in 5% of lesions in one study [10]. In another article, both the herald patch and the secondary lesions of PR displayed white scales [11]. Brown pigmented scales were seen clinically and dermoscopically in our study and are found frequently in our population as a result of serum and some amount of melanin present in the stratum corneum [4]. Brown dots were present in 55% of lesions of PR patients along with diffuse brown pigmentation in 25% patients. (Figure 3C) The brown dots corresponding to the central crusts are due to spongiotic tissue reaction plus hemosiderin deposits from extravasated red blood cells and lymphocytic infiltrate [12]. Diffuse brown pigmentation seen in older lesions corresponding to post-inflammatory hyper-pigmentation can be attributed to the dark skin of the study subjects. Thus, lesions of PR generally have a red background with unspecific distribution of dotted blood vessels. Scaling can be either white in color or brown pigmented with a patchy pattern, present either centrally or peripherally. Early lesions generally have central scaling with brown dots owing to the spongiotic reaction seen in PR, whereas the older lesions have peripheral scaling with diffuse brown pigmentation corresponding to post-inflammatory hyperpigmentation. While a combination of dotted blood vessels in a uniform distribution over a red background with overlying white scales in a diffuse pattern was highly specific for the diagnosis of GP, with progression of disease, the distance between the dotted blood vessels increases. Therefore, the dermoscopic differences between GP and PR found in this study reflect the known underlying histopathological features.

### Limitations

This study employs a very small sample. The evolution of lesions could not be observed, as this was a cross-sectional study. More studies with a larger sample size are required in the future.

## Conclusions

Patients with GP have a uniform arrangement of dotted blood vessels over a dull or bright red background with overlying diffuse white scaling. Patients with PR have dotted blood vessels in an unspecific fashion with overlying brown pigmented or white scales in peripheral or central regions, depending on the stage of the disease. Dermoscopy can also be used to study the evolution of disease in both GP and PR, although further studies are needed to confirm the same.

## Figures and Tables

**Table 1:** Characterstics of Vessels in Dermoscopic Lesions in Both Guttate Psoriasis and Pityriasis Rosea

Dermoscopic Features of Lesions		Guttate Psoriasis	Pityriasis Rosea	Statistical Significance (P Value)

**Vessel Characteristics**				

Vessel distribution	Uniform	17(85%)	1(5%)	P < 0.05
	Unspecific	3(15%)	12(60%)	

Vessel morphology	Red dots	20 (100%)	13 (65%)	P < 0.05

Blood vessels absent in		0	7(35%)	

**Background Color**				

Bright red		11(55%)	7(35%)	P = 0.068 (> 0.05)

Dull red		9(45%)	7(35%)	

Hyperpigmented background color		0	2(10%)	

Skin color		0	4(20%)	

**Scale Color**				

White		20 (100%)	11 (55%)	P < 0.05

Brown pigmented		0	9 (45%)	

**Scale Pattern**				

Diffuse		18(90%)	2(10%)	P < 0.05

Patchy		0	4(20%)	

Peripheral		2(10%)	7(35%)	

Central		0	7(35%)	

**Pigment Pattern**				

Brown dots		0	11(55%)	P < 0.05

Diffuse brown pigmentation		0	5(25%)	

Pigment absent		20(100%)	4(20%)	

**Table 2:** Dermoscopic and Histopathological Correlation of Guttate Psoriasis

Guttate Psoriasis	
Dermoscopic Features	Histopathological Features
Uniform distribution of red dots	Dilated capillaries in regularly elongated dermal papillae with suprapapillary thinning
Diffuse white scaling	More uniform parakeratosis (more confluent)
Red background	Vascular dilatation

**Table 3:** Dermoscopic and Histopathological Correlation of Pityriasis Rosea

Pityriasis Rosea	
Dermoscopic Features	Histopathological Features
Unspecific distribution of red dots	Non uniform dilatation of capillaries in the dermal papillae
Patchy scaling	Focal parakeratosis
Red background	Vascular dilatation
Brown dots	Spongiotic tissue reaction plus hemosiderin deposits from extravasated red blood cells and lymphocytic infiltrate
